# Effects of E-Liquid Formulations on Nicotine Vapor Pressure and Implications for Nicotine Delivery and Toxicity

**DOI:** 10.3390/toxics14050433

**Published:** 2026-05-14

**Authors:** Kaiyuan Wang, Xue Gong

**Affiliations:** 1 School of Aeronautics and Astronautics, Sun Yat-sen University, Shenzhen 518107, China; 2 Vehicle Measurement, Control and Safety Key Laboratory of Sichuan Province, School of Automobile and Transportation, Xihua University, Chengdu 610039, China

**Keywords:** nicotine vapor pressure, nicotine delivery, e-cigarettes, e-liquids, volatility

## Abstract

Electronic cigarettes are widely used as alternatives to conventional cigarettes. However, the relationships among e-liquid formulation, nicotine volatility, and nicotine delivery remain insufficiently investigated due to limited data on nicotine vapor pressure in e-liquid systems. This study aimed to investigate the effects of e-liquid formulations on nicotine vapor pressure, explore the underlying mechanisms, and establish correlations with nicotine delivery and pharmacokinetics. A headspace method was applied to measure nicotine vapor pressure at 37 °C, with variables including nicotine concentration, PG/VG ratio, organic acid type and ratio, and water content. The results showed that nicotine vapor pressure increased linearly with free-base nicotine fraction and decreased monotonically with increasing PG/VG ratio, acid-to-nicotine molar ratio, and water content. In addition, nicotine vapor pressure followed the order: free-base e-liquids > nicotine salt e-liquids > conventional cigarettes. Then, a correlation analysis was conducted between nicotine vapor pressure and nicotine pharmacokinetics. Lower vapor pressure correlated with deeper lung deposition, higher plasma nicotine, and greater potential toxicity, while higher vapor pressure correlated with more deposition in the upper respiratory tract and potential local irritation. Overall, nicotine vapor pressure can serve as an indicator for predicting nicotine delivery, supporting the rational regulation of e-liquid formulations and health risk assessment.

## 1. Introduction

Electronic cigarettes (e-cigarettes) have emerged over the past decade as one of the most popular alternatives to traditional combustible cigarettes [1,2,3]. According to the World Health Organization (WHO) [4], the global number of current e-cigarette users has exceeded 100 million, with adolescent usage prevalence being nine times higher than that among adults. Most commercial e-cigarettes contain nicotine, a highly addictive substance that is also harmful to human health. Nicotine exposure not only leads to long-term dependence and withdrawal symptoms [5,6,7], but also causes adverse effects on the cardiovascular, respiratory, and nervous systems [8,9,10]. Therefore, it is critically important and urgent to systematically investigate the release, delivery, and related health risks of nicotine in e-cigarette products.

In e-cigarettes, nicotine is initially dissolved in the e-liquid, which typically comprises propylene glycol (PG), vegetable glycerin (VG), organic acids, and flavoring compounds as key formulation components [11,12]. Upon activation of an e-cigarette, the e-liquid is heated, vaporized, and atomized into an inhalable aerosol. During this process, nicotine is dispersed within the aerosol droplets and partitions between the vapor and liquid phases [13,14,15]. After inhalation, these nicotine-containing aerosols enter the respiratory tract, where nicotine is gradually released from the aerosol droplets and subsequently deposited on the surfaces of the respiratory tract and alveoli prior to systemic absorption [16,17,18]. Throughout the entire release and transport process, nicotine continuously undergoes phase partitioning between the vapor and liquid phases. This partitioning behavior indicates that nicotine volatility is a key physicochemical property governing its delivery efficiency and subsequent biological effects.

Previous studies have confirmed that nicotine volatility significantly affects nicotine delivery efficiency from e-cigarettes to users [19,20]. Higher volatility promotes nicotine transfer into the vapor phase, which substantially alters its deposition sites within the respiratory tract. Free-base nicotine, with higher volatility, tends to vaporize rapidly and deposit more in the upper respiratory tract, leading to enhanced upper respiratory deposition [21,22,23]. In contrast, protonated nicotine exhibits lower volatility and remains mainly within aerosol droplets, allowing deeper penetration into the lung and thereby enhancing deep lung delivery [24]. Moreover, e-liquid composition and pH can affect nicotine volatility, resulting in obvious variations in nicotine delivery efficiency [25,26,27]. These findings demonstrate that lower nicotine volatility is associated with greater nicotine deposition in the deep lung, as well as increased addictive and toxicological potential.

The volatility of nicotine can be quantitatively characterized by its vapor pressure. The vapor pressure of pure nicotine has been measured in previous studies [28,29,30], reporting values of approximately 2.7 to 5.7 Pa at 25 °C and a marked increase with rising temperature. As for e-cigarettes and e-liquids, only a limited number of studies have focused on determining the actual vapor pressure of nicotine. Pankow et al. [31] provided gas/particle partitioning constants and vapor pressure values of nicotine in 50/50 PG/VG solutions. St Charles and Moldoveanu [32] experimentally investigated the partial vapor pressures, activity coefficients, and Henry volatility of nicotine in binary mixtures with PG and with VG at 298.15 K using a headspace method. However, nicotine vapor pressure is strongly affected by various e-liquid formulation parameters, including PG/VG ratio, organic acid type, acid-to-nicotine ratio, and water content, which have not been fully investigated. Moreover, in-depth analyses of how these parameters influence nicotine volatility and its correlation with nicotine delivery are still insufficient. Therefore, further research is needed to acquire nicotine vapor pressure data for diverse e-liquid formulations and reveal the key factors governing nicotine volatility and delivery.

This study presents a comprehensive investigation of nicotine vapor pressure in different e-liquid formulations, along with an assessment of its impacts on nicotine delivery. First, the basic theory of nicotine vapor pressure is described, and a headspace experimental method is established for vapor pressure measurement. Subsequently, the measured nicotine vapor pressure data and corresponding analyses are presented. Effects of nicotine mass fraction, PG/VG ratio, organic acid type and ratio, and water content on nicotine vapor pressure, as well as cross-comparisons between e-liquids and conventional cigarettes, are illustrated. Furthermore, the underlying mechanisms of nicotine volatility are explained in detail by the chemical form of nicotine and molecular interactions between nicotine and other components. Finally, nicotine delivery is analyzed based on the vapor pressure data from this study and the pharmacokinetic data obtained from the literature [33]. This analysis is further correlated with toxicological implications to enable a comprehensive evaluation of different nicotine-containing products.

## 2. Materials and Methods

### 2.1. Theory of Nicotine Vapor Pressure

For pure liquid nicotine, its volatility can be quantitatively described by the saturated vapor pressure ($p_{nic,s}$), defined as the equilibrium vapor pressure of pure liquid nicotine at a specified temperature. Under this equilibrium condition, the rate of nicotine molecules evaporating from the liquid phase equals their condensation rate from the vapor phase. $p_{nic,s}$ is an intrinsic physicochemical property of pure nicotine at a constant temperature.

In e-liquid systems, the actual vapor pressure of nicotine corresponds to its partial pressure ($p_{nic}$), which differs greatly from $p_{nic,s}$. This partial pressure is governed primarily by two key factors: the nicotine concentration in the e-liquid and the presence of other e-liquid components (e.g., PG, VG, organic acids, and water). The dependence of nicotine partial pressure on its concentration follows Raoult’s law, which states that the partial pressure of a component is proportional to its mole fraction in the liquid phase and its saturated vapor pressure in the pure state. The influence of other e-liquid components is considered by an equivalent activity coefficient, γ. Therefore, the vapor pressure of nicotine in e-liquids can be expressed as

(1) $$p_{nic}=p_{nic,s}x_{nic}\gamma ,$$

where $x_{nic}$ denotes the mole fraction of nicotine in the e-liquid.

The activity coefficient γ accounts for deviations of the e-liquid system from an ideal solution. Intermolecular interactions between nicotine and other e-liquid components modify the escaping tendency of nicotine molecules from the liquid phase, thereby affecting its actual partial pressure. In typical PG/VG-based systems, $x_{nic}$ is much smaller than 1 and γ is greater than 1, so the actual vapor pressure of nicotine in e-liquids is lower than $p_{nic,s}$. Notably, temperature effects on nicotine vapor pressure were not investigated in this study. All experiments were conducted at a constant temperature of 37 °C, which represents human body temperature relevant to e-cigarette aerosol transport and respiratory deposition after inhalation. Although the heating temperature can reach 200–300 °C during e-liquid atomization, inhaled e-cigarette aerosol mixtures rapidly equilibrate with the body temperature in the oral cavity, as demonstrated by Asgharian et al. [34]. Therefore, 37 °C is the most relevant temperature governing nicotine delivery efficiency in humans.

### 2.2. Headspace Measurement and Vapor Pressure Determination

Headspace sampling coupled with gas chromatography-mass spectrometry (GC-MS) was employed to determine the partial vapor pressure of nicotine in e-liquids. This method has been widely used in previous studies for the characterization and quantification of volatile substances [35,36,37]. All e-liquid samples were sealed in headspace vials, with one half filled by the e-liquid and the other half by the gas phase. These vials were incubated in a thermostatic water bath at 37 °C for 24 h to establish thermodynamic equilibrium between the liquid and vapor phases. After equilibration, the headspace gas was sampled using a sampling probe and injected into an Agilent 7890A/5975C GC-MS system (Santa Clara, CA, USA) for quantitative analysis. The sampling process had negligible effects on the phase equilibrium. The GC system was equipped with a DB-Waxetr capillary column (30 m × 0.25 mm id, 0.25 μm film thickness) suitable for the separation, while the MS detector was operated in selected ion monitoring (SIM) mode to ensure high sensitivity and selectivity for nicotine. Helium was used as the carrier gas at a constant flow rate of 1.0 mL/min. The GC oven temperature program was set as follows: initial temperature 50 °C (hold 1.0 min), ramped to 240 °C at 10 °C/min, and held for 10 min. The injector temperature, MS ion source temperature, and auxiliary temperature were set at 280 °C, 230 °C, and 250 °C, respectively.

The nicotine concentration in the headspace vapor, determined by GC-MS, was further converted to nicotine partial pressure using the ideal gas law:

(2) $$p_{nic}=c_{g}RT,$$

where $c_{g}$ is the molar concentration of nicotine in the vapor phase, R is the universal gas constant, and T is the absolute temperature (310.15 K). Using this approach, the nicotine vapor pressure for each e-liquid formulation was quantitatively derived from the measured headspace concentration.

By combining the theoretical vapor pressure equation, Equation (1), with the experimental vapor pressure expression, Equation (2), the overall activity coefficient γ of nicotine in e-liquids can be quantitatively determined as

(3) $$\gamma =c_{g}RT/(p_{nic,s}x_{nic}),$$

Using the derived activity coefficient together with Equation (1), the nicotine vapor pressure under a wide range of formulation conditions can be further predicted. This approach allows the calculation of nicotine vapor pressure beyond the measured experimental data points in this study.

### 2.3. E-Liquid Sample Preparation

A series of e-liquid samples with varying compositions were prepared to investigate the effects of different formulation factors on nicotine vapor pressure. The controlled variables included nicotine mass fraction, PG/VG mass ratio, organic acid type (benzoic acid and lactic acid), acid-to-nicotine molar ratio, and water mass fraction. The specific values of these variables for all e-liquid samples are listed in Table 1. The formulation parameters in this table were set according to typical e-liquid specifications. Conversions between mass and molar quantities can be easily performed based on the corresponding molecular weights.

For e-liquid preparation, all components were accurately weighed and thoroughly mixed. The mixtures were then continuously stirred in a thermostatic water bath at 60 °C for a sufficient duration to ensure complete dissolution and homogeneity. After cooling to 37 °C, each e-liquid sample was divided into three equal aliquots, transferred into individual headspace vials, and sealed immediately for subsequent headspace analysis. Accordingly, all measurements were performed with three parallel samples (*N* = 3) for each condition.

To facilitate comparative analysis, mainstream tobacco products were also included as reference samples, namely Chunghwa cigarettes and 1R6F reference cigarettes. Two sets of headspace measurements were carried out for these tobacco samples. In the first set, cut tobacco was placed directly into headspace vials to characterize the intrinsic nicotine volatility of the tobacco substance. In the second set, mainstream aerosol was generated using a commercial smoking machine manufactured by Huironghe (Beijing, China) and collected on Cambridge filters under the ISO 3308 standard puffing regimen (puff volume: 35 mL, puff duration: 2 s, puff interval: 60 s). After 10 puffs, the Cambridge filter loaded with aerosol matter was immediately sealed in vials to determine the nicotine vapor pressure in the headspace. The headspace protocol was slightly different with a longer equilibration time (120 h) applied for solid tobacco samples to ensure sufficient nicotine release from the solid matrix. Other measurement procedures remained the same as those used for e-liquid samples. Following the same principle as for e-liquids, the vapor phase in the sealed vials eventually reached dynamic equilibrium with the tobacco pieces or aerosols, allowing quantification of nicotine partial pressure for tobacco products.

## 3. Results

This section presents the experimentally measured nicotine vapor pressure data for the e-liquid formulations listed in Table 1 and cigarette samples. The results are organized and discussed in sequence according to the formulation variables investigated, including nicotine mass fraction, PG/VG mass ratio, organic acid type and molar ratio, water content, and cross-product comparison.

### 3.1. Effects of Nicotine Mass Fraction on Nicotine Vapor Pressure

**Figure 1:**
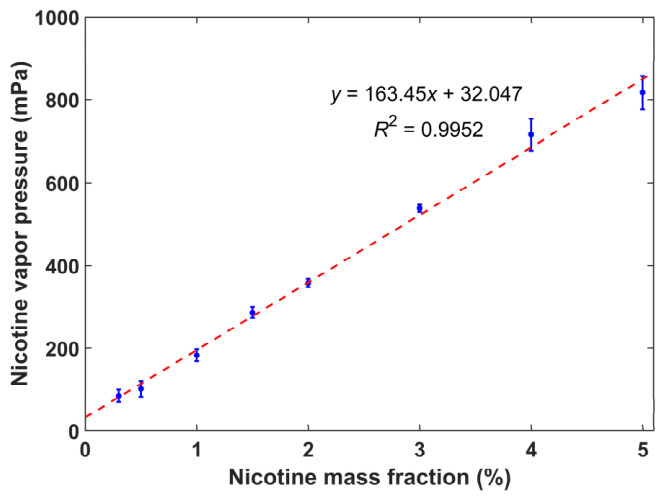
Variation in nicotine vapor pressure with nicotine mass fraction. Blue symbols represent experimental data, and the red dashed line represents the fitted curve.

Figure 1 shows the relationship between nicotine vapor pressure and its mass fraction. The scattered points accompanied by error bars represent the experimentally measured data, while the red dashed line represents the linear regression fit. As seen in the figure, the nicotine vapor pressure increases almost linearly with increasing nicotine mass fraction. The linear regression equation is *y* = 163.45*x* + 32.047, and the coefficient of determination *R*^2^ is 0.9952. Such a high *R*^2^ value indicates a strong linear correlation between nicotine vapor pressure and nicotine mass fraction. This near-perfect linear correspondence agrees well with Raoult’s law, which further validates the reliability and accuracy of the present measurements for nicotine vapor pressure. The above results demonstrate that the nicotine mass fraction is a dominant factor governing nicotine vapor pressure in e-liquids.

### 3.2. Effects of PG/VG Ratio on Nicotine Vapor Pressure

**Figure 2:**
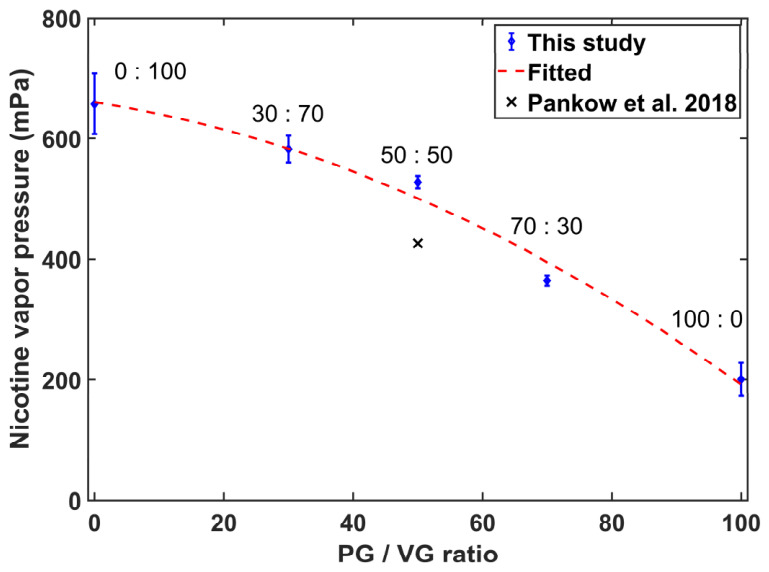
Variation in nicotine vapor pressure with PG/VG ratio [31].

Figure 2 illustrates the variation in nicotine vapor pressure with the PG/VG ratio in e-liquids. For comparison, the black cross symbol represents the calculated value for the 50:50 PG/VG system using parameters reported by Pankow et al. [31]. The experimental data are in reasonable agreement with the literature value, and the observed difference lies within the acceptable range for nicotine vapor pressure studies. This agreement further confirms the reliability of the present headspace measurements. As the PG/VG ratio increases from 0:100 to 100:0, nicotine vapor pressure decreases monotonically over the entire range. The nicotine vapor pressure reaches a maximum of 657 mPa at a PG/VG ratio of 0:100 (pure VG) and decreases to a minimum of 200 mPa at 100:0 (pure PG). This negative correlation indicates that the PG/VG ratio is a critical factor influencing nicotine volatility in e-liquids, with a higher PG proportion corresponding to a lower nicotine vapor pressure. The monotonic decrease can be attributed to the intermolecular interactions between PG/VG and nicotine. Since PG exerts stronger solvation effects on nicotine than VG, the enhanced intermolecular forces between PG and nicotine reduce the volatility of nicotine.

### 3.3. Effects of Organic Acids on Nicotine Vapor Pressure

**Figure 3:**
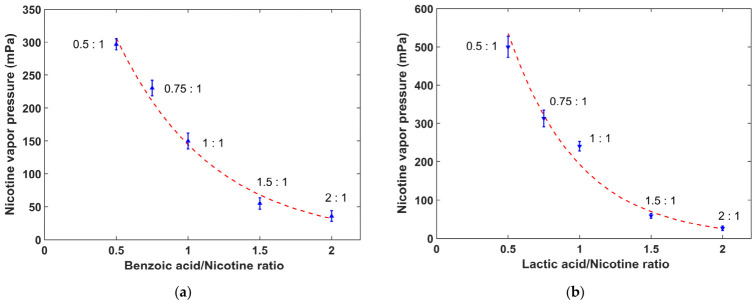
Variations in nicotine vapor pressure with acid-to-nicotine molar ratio: (**a**) Nicotine benzoate formulations; (**b**) Nicotine lactate formulations.

Figure 3a,b illustrate the effects of benzoic acid and lactic acid on nicotine vapor pressure in e-liquids, respectively. For both organic acids, nicotine vapor pressure decreases monotonically as the acid-to-nicotine molar ratio increases from 0.5:1 to 2:1. Specifically, nicotine benzoate formulations show a maximum nicotine vapor pressure of approximately 297 mPa at a ratio of 0.5:1 and a minimum of around 36 mPa at 2:1. In comparison, nicotine lactate formulations exhibit a maximum nicotine vapor pressure of approximately 500 mPa at a ratio of 0.5:1 and a minimum of about 26 mPa at 2:1. The underlying mechanism for these negative correlations can be attributed to the protonation of free-base nicotine by benzoic and lactic acids, which converts volatile nicotine into non-volatile nicotine salts and thus suppresses nicotine evaporation. Furthermore, at the same acid-to-nicotine ratio, nicotine vapor pressure is higher in nicotine lactate formulations than in nicotine benzoate formulations, indicating a weaker suppression effect of lactic acid on nicotine evaporation compared to benzoic acid. These results indicate that organic acid type and acid-to-nicotine ratio are key factors affecting nicotine volatility.

### 3.4. Effects of Water Content on Nicotine Vapor Pressure

**Figure 4:**
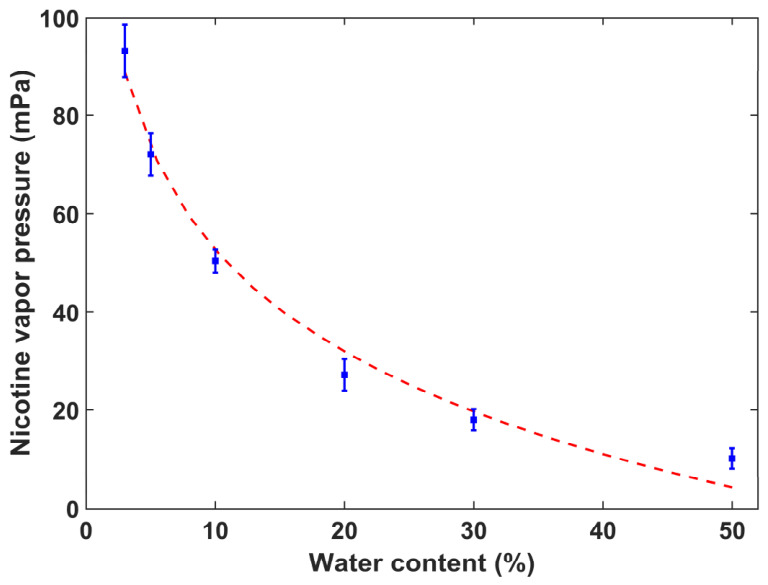
Variation in nicotine vapor pressure with water content.

Figure 4 shows the variation in nicotine vapor pressure with water content in e-liquids. The data points with error bars represent the experimental measurements, while the red dashed line indicates the fitted trend. As water content increases from approximately 3% to 50%, nicotine vapor pressure decreases monotonically. The nicotine vapor pressure reaches a maximum of about 93 mPa at 3% water content and declines to a minimum of around 10 mPa at 50% water content. This strong negative correlation demonstrates that water content is also a key factor influencing nicotine volatility, with higher water content leading to a substantial reduction in nicotine vapor pressure. The underlying mechanism can be attributed to the enhanced hydrogen bonding interactions between nicotine and water molecules, which suppress nicotine evaporation from the liquid phase.

### 3.5. Comparison of Nicotine Vapor Pressure Between E-Liquids and Cigarettes

**Figure 5:**
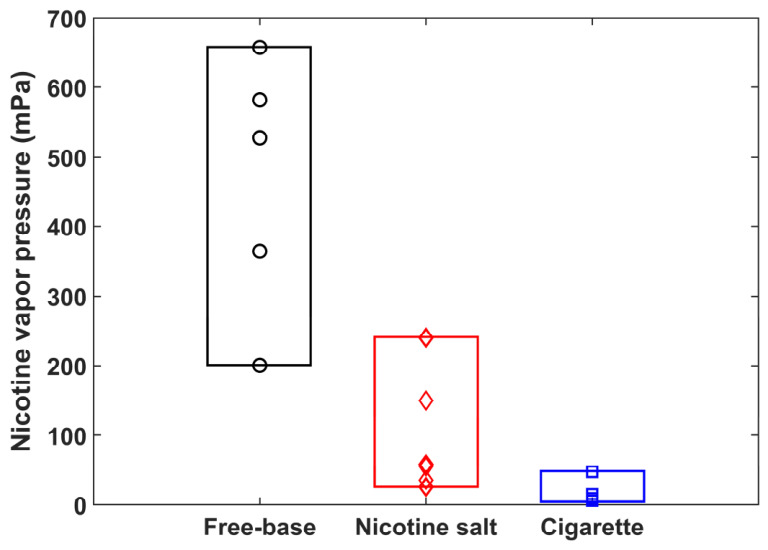
Comparison of nicotine vapor pressure among free-base nicotine e-liquids, nicotine salt e-liquids, and conventional cigarettes.

Figure 5 presents a box plot comparison of nicotine vapor pressure across three sample types: free-base nicotine e-liquids, nicotine salt e-liquids, and conventional cigarettes. The vapor pressure data were obtained from Section 3.2 for the free-base nicotine e-liquids and Section 3.3 for the nicotine salt e-liquids with an acid-to-nicotine molar ratio ≥ 1. Both types of e-liquids had an identical nicotine mass fraction of 3% for direct comparison. The cigarette group integrated the datasets from both cut tobacco and mainstream aerosol samples, with averaged nicotine vapor pressure values of 5.9 mPa (Chunghwa) and 9.3 mPa (1R6F) for cut tobacco, and 15.3 mPa (Chunghwa) and 47.5 mPa (1R6F) for mainstream aerosol. The free-base nicotine formulations exhibit the highest nicotine vapor pressure, with values ranging from approximately 200 to 657 mPa. In contrast, nicotine salt formulations show significantly lower vapor pressure with values ranging from approximately 26 to 240 mPa, while conventional cigarettes display the lowest vapor pressure, ranging from approximately 6 to 48 mPa. These results clearly demonstrate that the nicotine vapor pressure follows the order: free-base nicotine > nicotine salt > conventional cigarette, highlighting the strong suppression effect of protonation in nicotine salts and solid-phase binding effects in conventional cigarettes on nicotine volatility.

## 4. Discussion

### 4.1. Effects of E-Liquid Composition on Nicotine Vapor Pressure

Based on the experimental results presented in Section 3, the composition of e-liquids was confirmed to significantly affect nicotine vapor pressure, with each formulation variable exhibiting a distinct quantitative effect on nicotine volatility.

For free-base nicotine e-liquids, nicotine vapor pressure showed a linear positive correlation with the nicotine mass fraction, which is consistent with Raoult’s law and validates the reliability of the present experimental measurements. The PG/VG ratio, a fundamental solvent composition parameter of e-liquids, exhibited a notable negative correlation with nicotine vapor pressure. The measured nicotine vapor pressure at 50:50 PG/VG ratio was consistent with the value calculated using parameters reported by Pankow et al. [31], which further supports the reliability of the present measurements. In addition, nicotine vapor pressure decreased monotonically with increasing PG fraction, and the pure PG system yielded the minimum nicotine vapor pressure while the pure VG system showed the maximum. These observations for the pure PG and pure VG systems are consistent with the results reported by St Charles and Moldoveanu [32].

For nicotine salt e-liquids, benzoic acid and lactic acid were used as organic acid additives, and both induced a monotonic decrease in nicotine vapor pressure with increasing acid-to-nicotine molar ratio. The type of organic acid also led to obvious differences in nicotine volatility, with nicotine vapor pressure in nicotine lactate formulations higher than that in nicotine benzoate formulations. Water content, as an important auxiliary component, caused a continuous and substantial reduction in nicotine vapor pressure when increased from 3% to 50%.

These results indicate that nicotine vapor pressure in e-liquids can be regulated by adjusting the free-base nicotine mass fraction, PG/VG ratio, type and proportion of organic acids, and water content. It should be noted that e-liquid compositions are highly complex and include various other additives such as flavorings and fragrances. Their potential impacts on nicotine volatility were not explored in the present study and can be further investigated in future work.

### 4.2. Underlying Mechanisms of Nicotine Vapor Pressure

**Figure 6:**
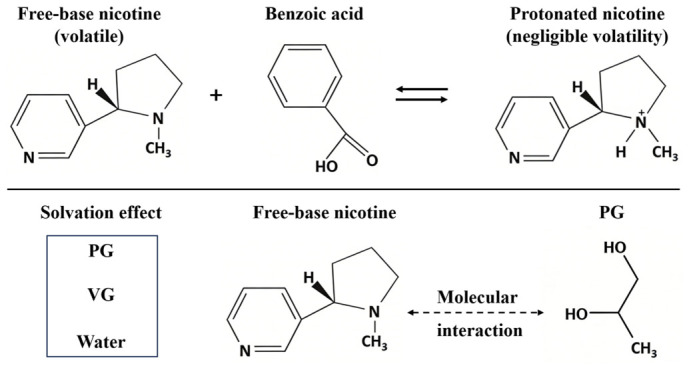
Schematic diagram of the reversible equilibrium between free-base nicotine and protonated nicotine, and the solvation interactions between nicotine and e-liquid solvents.

The variations in nicotine vapor pressure induced by different e-liquid formulations and conventional cigarettes can be fundamentally explained by the chemical form of nicotine and the molecular interactions between nicotine and other components. Figure 6 shows a schematic diagram illustrating the reversible equilibrium between free-base nicotine and protonated nicotine, as well as the solvation interactions between nicotine and e-liquid solvents.

Nicotine exists in two main chemical forms in e-liquids: the free-base form and the protonated ionic form. The relative proportion of these two forms is the fundamental factor determining nicotine volatility. Free-base nicotine is the primary volatile form of nicotine in e-liquids, since unprotonated molecules exhibit weak intermolecular interactions with the liquid phase, directly leading to the high nicotine vapor pressure observed in free-base e-liquids. As shown in Figure 1, the nearly perfect linear positive correlation between the free-base nicotine fraction and vapor pressure reflects that a higher content of volatile free-base nicotine directly increases vapor pressure.

In nicotine salt e-liquids containing organic acids, the carboxylic acid groups of benzoic acid and lactic acid react with the basic nitrogen atom of nicotine to trigger a protonation reaction. In this process, hydrogen ions are transferred from organic acids to nicotine molecules, converting volatile free-base nicotine into non-volatile protonated nicotine salts. As the acid-to-nicotine molar ratio increases, a larger proportion of free-base nicotine is converted to the protonated form, thereby causing a monotonic decrease in nicotine vapor pressure, as shown in Figure 3. The different effects of benzoic acid and lactic acid on nicotine volatility originate from their differences in acid strength and molecular structure. Benzoic acid achieves a higher degree of nicotine protonation at the same acid-to-nicotine molar ratio, which results in lower nicotine vapor pressure in nicotine benzoate formulations than in nicotine lactate ones.

PG and VG are the main organic solvents in e-liquids and modify nicotine volatility through distinct solvation effects and intermolecular interactions. Both solvents form intermolecular forces with nicotine, but PG exerts a stronger solvation effect due to its more polar molecular structure and higher hydrogen-bonding capacity. In contrast, VG shows a relatively weak solvation effect, allowing free-base nicotine to volatilize more easily and resulting in higher vapor pressure in pure VG systems than in pure PG systems, as shown in Figure 2. Water acts as an auxiliary polar component in e-liquids and forms strong hydrogen bonds with nicotine molecules. These hydrogen bonds suppress the escape of nicotine molecules into the gas phase and thus cause a substantial reduction in nicotine vapor pressure with increasing water content, as shown in Figure 4.

The significant difference in nicotine vapor pressure between e-liquids and conventional cigarettes arises from the distinction between liquid-phase solvation and solid-phase binding. Unlike e-liquids, in which nicotine is dispersed in a liquid solvent, nicotine in conventional cigarettes is tightly bound to complex solid tobacco matrices. This solid-phase binding creates a stable adsorption system between nicotine and tobacco, which strongly restricts nicotine evaporation. Consequently, only a small amount of nicotine can escape from the solid tobacco, leading to lower nicotine vapor pressure in conventional cigarettes than in free-base and nicotine salt e-liquids, as presented in Figure 5.

### 4.3. Correlations with Nicotine Delivery and Pharmacokinetics

Nicotine vapor pressure is not only a key physicochemical property governing nicotine volatility, but also exhibits a quantifiable correlation with the respiratory deposition behavior, delivery efficiency, and subsequent pharmacokinetic (PK) profiles of nicotine. A theoretical derivation explains this relationship: the higher the nicotine vapor pressure, the more nicotine molecules released into the gas phase and deposited in the upper respiratory tract upon inhalation, which consequently results in reduced deposition in the lungs and lower plasma nicotine concentrations. In contrast, nicotine with lower vapor pressure deposits less in the upper respiratory tract, allowing more nicotine to penetrate the deep lung and thus leading to higher plasma nicotine levels. Therefore, the deposition pattern and delivery efficiency of nicotine are directly reflected in the nicotine PK characteristics. The maximum plasma nicotine concentration ($C_{max}$) is a key indicator of nicotine delivery efficiency and biological effects [38].

**Figure 7:**
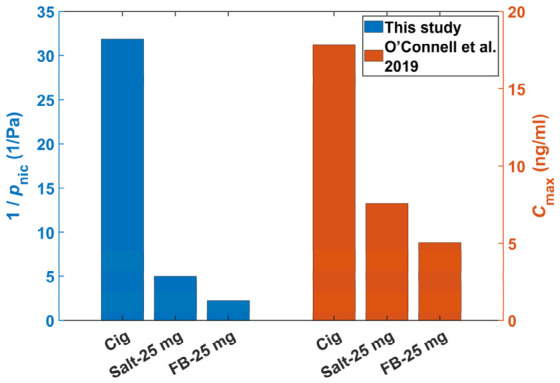
Relationship between the inverse of nicotine vapor pressure ($1/p_{nic}$, left axis) and the maximum plasma nicotine concentration [33] ($C_{max}$, right axis) across three product types: conventional cigarettes (Cig), nicotine salt e-liquids (Salt-25 mg), and free-base nicotine e-liquids (FB-25 mg).

Figure 7 presents a dual-Y-axis bar chart comparing the inverse of nicotine vapor pressure ($1/p_{nic}$, left axis) and the maximum plasma nicotine concentration ($C_{max}$, right axis) across three product types: conventional cigarettes (Cig), nicotine salt e-liquids (Salt-25 mg), and free-base nicotine e-liquids (FB-25 mg). The notation 25 mg indicates a nicotine concentration of 25 mg per milliliter in the e-liquids. The nicotine vapor pressure data were obtained from the experimental measurements and fitted equations in the present study, while the pharmacokinetic data were taken from the published literature [33]. Although the e-liquid formulations differed between the two studies, the key variables remained comparable. As shown in the figure, cigarettes exhibit the highest $1/p_{nic}$ value, indicating the weakest nicotine volatility, and also display the highest $C_{max}$, representing the highest nicotine delivery. In contrast, free-base nicotine e-liquids (FB-25 mg) show the lowest $1/p_{nic}$ and $C_{max}$ values, corresponding to the strongest volatility and lowest delivery efficiency. Nicotine salt e-liquids (Salt-25 mg) exhibit intermediate values for both parameters. These results demonstrate a strong positive correlation between $1/p_{nic}$ (a measure of nicotine retention) and $C_{max}$ (a measure of plasma delivery efficiency), indicating that lower nicotine vapor pressure corresponds to higher plasma nicotine delivery.

These findings are well aligned with viewpoints established in previous pharmacokinetic studies [39,40]. For instance, lower nicotine volatility may favor greater lung deposition and absorption over buccal uptake, enabling more rapid and efficient nicotine delivery to the brain. While previous studies inferred such relationships indirectly from pharmacokinetic profiles, the present study provides direct quantitative evidence by experimentally quantifying nicotine vapor pressure. The consistent trend observed among vapor pressure, deposition behavior, and plasma nicotine concentration further supports the mechanistic link between $1/p_{nic}$ and $C_{max}$.

### 4.4. Implications for Nicotine Toxicity in E-Cigarettes

Nicotine exposure level forms the basis for evaluating its acute and chronic toxic effects as well as its addictive potential. In principle, nicotine concentration represents the total nicotine content in e-liquids and serves as a fundamental parameter for nicotine exposure. In this study, the strong correlations between nicotine vapor pressure and plasma nicotine concentration indicate that nicotine vapor pressure reflects the delivery efficiency of nicotine from the aerosol phase to the human body. Therefore, combining these two parameters allows a comprehensive and quantitative assessment of actual nicotine exposure levels associated with e-liquid and tobacco products.

For conventional cigarettes, the combination of high nicotine concentration and extremely low nicotine vapor pressure leads to high plasma nicotine concentrations and large nicotine exposure doses upon inhalation. This directly increases the risk of acute toxic effects (e.g., cardiovascular perturbations, respiratory irritation, central nervous system excitation) and promotes the development of nicotine dependence.

For nicotine salt e-liquids, their toxicological profile is highly modifiable due to adjustable nicotine concentrations and tunable protonation degrees. The potential toxic and addictive risks of these products are closely related to their actual formulation parameters. Notably, nicotine salt e-liquids with high nicotine concentration and high protonation degree can achieve nicotine delivery efficiency and exposure levels approaching those of conventional cigarettes, resulting in high toxic and addictive potential. This analysis is consistent with a recent pharmacokinetic study [41], which reported that high-concentration nicotine salt e-liquids can provide nicotine delivery comparable to conventional cigarettes.

For free-base nicotine e-liquids, pulmonary delivery efficiency and systemic nicotine exposure are relatively low. However, owing to their high vapor pressure, large numbers of nicotine molecules deposit in the oral, pharyngeal, upper tracheal, and bronchial regions. This localized deposition pattern causes increased local nicotine exposure in these mucosal tissues, potentially inducing adverse effects including oral and pharyngeal mucosal irritation, chronic inflammation and epithelial damage. Therefore, a comprehensive risk assessment should be performed beyond the evaluation of systemic nicotine delivery alone.

The above analysis confirms that nicotine vapor pressure is not only a reliable indicator of nicotine delivery efficiency, but also a key indirect predictor of nicotine-related toxicological risk for tobacco and e-liquid products. Moreover, this parameter can be measured via headspace experiments or estimated from existing fitting equations, avoiding the need for complex and ethically constrained human trials. The present analysis provides a novel strategy for the comprehensive toxicological and health risk assessment of nicotine-containing products.

### 4.5. Limitations of This Study

This study provides fundamental physicochemical data on nicotine vapor pressure of e-liquids under controlled, standardized conditions at 37 °C. In real-world vaping situations, e-liquids undergo heating and atomization, and the chemical composition of vaped aerosols differs from that of unvaped e-liquids. For example, the aerosol nicotine concentration has been reported to be lower than the initial nicotine concentration in the e-liquid [42], which tends to yield a lower nicotine vapor pressure in the generated aerosol compared with that of unvaped e-liquid. Therefore, estimating respiratory deposition behavior based on unvaped e-liquid properties may not be fully accurate, which represents the primary limitation of this study. The discrepancy mainly arises from the transfer efficiency of each e-liquid component from unvaped e-liquids to vaped aerosols. Notably, transfer efficiencies of nicotine and flavor chemicals have been quantified under various device power settings and puffing topographies [43]. Correcting the component transfer efficiency would bring predictions closer to realistic aerosol conditions. Nevertheless, nicotine release during atomization is positively correlated with its initial concentration in unvaped e-liquids, so the use of unvaped e-liquids for preliminary deposition correlation is reasonable. Furthermore, the atomization process involves complex phase changes with uncontrollable variables (e.g., consistency of e-cigarette devices, the matching between device power and e-liquid formulations), which poses substantial challenges for acquiring standardized data and quantitative relationships. These challenges highlight the need for direct measurements of nicotine vapor pressure in vaped aerosols, which represents an important direction for future research.

In addition, the analysis and inference of aerosol dynamics and deposition presented in this study are preliminary. The primary aim is to illustrate the underlying logical relationship among nicotine vapor pressure, aerosol deposition, and plasma nicotine concentration. A more rigorous quantitative evaluation requires the establishment of a detailed aerosol deposition model that couples aerosol dynamics (e.g., coagulation, hygroscopic growth) with respiratory deposition dosimetry, which is the key focus of future research. Furthermore, the clearance mechanisms of deposited aerosol particles in the respiratory tract were not considered in the current analysis, and their integration into the deposition model will be an important topic for future study to provide a more complete assessment of nicotine retention and systemic exposure.

## 5. Conclusions

This study systematically investigated nicotine vapor pressure in e-liquids using a headspace-GC/MS method at 37 °C, focusing on the effects of key formulation variables including nicotine mass fraction, PG/VG ratio, organic acid type and molar ratio, and water content. The underlying mechanisms governing nicotine volatility were analyzed based on nicotine protonation and solvation effects. Cross-comparisons of nicotine vapor pressure among free-base e-liquids, nicotine salt e-liquids, and conventional cigarettes were performed, and the correlations between nicotine vapor pressure, respiratory deposition, nicotine delivery, and toxicological risks were also evaluated.

Experimental results demonstrated that nicotine vapor pressure increased linearly with the free-base nicotine mass fraction, and decreased monotonically with increasing PG/VG ratio, acid-to-nicotine molar ratio, and water content. Benzoic acid exhibited a stronger suppression effect on nicotine volatility than lactic acid owing to its greater protonation degree. The measured vapor pressure followed the order: free-base e-liquids > nicotine salt e-liquids > conventional cigarettes. In addition, a strong positive correlation was observed between $1/p_{nic}$ (a measure of nicotine retention) and $C_{max}$ (a measure of plasma delivery efficiency). Lower nicotine vapor pressure favored deep lung deposition and higher plasma nicotine, indicating enhanced systemic delivery, addiction potential, and acute toxicity. In contrast, free-base nicotine with high vapor pressure is likely to deposit less in the alveolar region and more in the upper respiratory tract, which may cause local mucosal irritation.

The present study verified that nicotine vapor pressure can serve as a reliable indicator for evaluating nicotine volatility and delivery efficiency, providing a practical tool for e-liquid formulation characterization and health risk assessment. Future research will focus on establishing a coupled physicochemical transport model to quantitatively simulate the phase partitioning, aerosol dynamics, and respiratory deposition processes of e-cigarette aerosols. Such a model will enable more precise prediction of nicotine delivery efficiency and biological outcomes.

## Figures and Tables

**Table 1:** Formulations of nicotine e-liquids used in the headspace measurements.

E-Liquid Formulations	Nicotine Mass Fraction	PG/VG Mass Ratio	Acid-to-Nicotine Molar Ratio	Water Mass Fraction
Free-base nicotine (variable concentration)	0.3%, 0.5%, 1%, 1.5%, 2%, 3%, 4%, 5%	50/50	-	-
Free-base nicotine (variable PG/VG ratio)	3%	0/100, 30/70, 50/50, 70/30, 100/0	-	-
Nicotine benzoate (variable acid-to-nicotine ratio)	3%	50/50	0.5:1, 0.75:1, 1:1, 1.5:1, 2:1	-
Nicotine lactate (variable acid-to-nicotine ratio)	3%	50/50	0.5:1, 0.75:1, 1:1, 1.5:1, 2:1	-
Nicotine benzoate (variable water content)	3%	50/50	1:1	3%, 5%, 10%, 20%, 30%, 50%

“-” indicates that the corresponding component or parameter was not included in the formulation.

## Data Availability

The original contributions presented in this study are included in the article. Further inquiries can be directed to the corresponding authors.
